# Interpretation of AI-Generated vs. Human-Made Images

**DOI:** 10.3390/jimaging11070227

**Published:** 2025-07-07

**Authors:** Daniela Velásquez-Salamanca, Miguel Ángel Martín-Pascual, Celia Andreu-Sánchez

**Affiliations:** 1 Máster Universitario en Contenidos de Comunicación Audiovisual y Publicidad (MUCAP), Universitat Autònoma de Barcelona, 08193 Barcelona, Spain; danielavelasquezsalamanca@gmail.com; 2 Research and Development, Radio Televisión Española Instituto, 08174 Barcelona, Spain; miguelangel.martin@rtve.es; 3 Neuro-Com Research Group, Universitat Autònoma de Barcelona, 08193 Barcelona, Spain

**Keywords:** image interpretation, AI-generated images, human-made images

## Abstract

AI-generated content has grown significantly in recent years. Today, AI-generated and human-made images coexist across various settings, including news media, social platforms, and beyond. However, we still know relatively little about how audiences interpret and evaluate these different types of images. The goal of this study was to examine whether image interpretation is influenced by the origin of the image (AI-generated vs. human-made). Additionally, we aimed to explore whether visual professionalization influences how images are interpreted. To this end, we presented 24 AI-generated images (produced using Midjourney, DALL·E, and Firefly) and 8 human-made images to 161 participants—71 visual professionals and 90 non-professionals. Participants were asked to evaluate each image based on the following: (1) the source they believed the image originated from, (2) the level of realism, and (3) the level of credibility they attributed to it. A total of 5152 responses were collected for each question. Our results reveal that human-made images are more readily recognized as such, whereas AI-generated images are frequently misclassified as human-made. We also find that human-made images are perceived as both more realistic and more credible than AI-generated ones. We conclude that individuals are generally unable to accurately determine the source of an image, which in turn affects their assessment of its credibility.

## 1. Introduction

In recent years, generative artificial intelligence has emerged as a highly relevant topic, widely covered by the media, alongside the launch of various tools, including generative adversarial networks (GANs) and variational autoencoders (VAEs). In particular, it is considered that artificial intelligence (AI) has established itself as a fundamental tool in the creative industries [1], paving the way for content creation through a simple instruction (prompt).

Image interpretation has a long-standing history in the field of AI, particularly with applications in medical contexts [2]. It has reshaped medicine, improving the experiences of both clinicians and patients [3], creating great opportunities to automate part of the complex process of accurately interpreting medical images [4]. So, several studies have proved that good AI models can outperform human-made visuals in several contexts, such as in clinical diagnosis in radiology [5]; in marketing across the quality, realism, and esthetics dimensions [6]; or in product design [7], among others.

However, being capable of distinguishing between AI-generated and human-made content is a different thing and is of great interest today [8]. Many studies have been developed to design AI algorithms capable of distinguishing between AI and non-AI creations [9]. In fact, there are quite good commercial detectors that perform really well, obtaining a high accuracy of over 98% [10].

In a related matter, expertise and professionalization have previously been linked to better performance in several areas, such as music [11,12], sports [13], driving [14,15], and media [16,17,18,19], among others. When comparing human artists to AI tools in the task of evaluating AI-generated images, human artists outperform the machines in certain specialized tasks [10]. In this context, the question is whether visual experts outperform non-experts in detecting or evaluating AI-generated images.

On another note, AI-generated images are becoming increasingly realistic. Most AI models promise more realistic representations with each new version released [20], even if most of these are biased towards white, Western realities [20,21]. Meanwhile, the widespread use of ‘fake’ images contributes to a growing difficulty in discerning what is real from what is fabricated [22]. This has become a concern regarding the vulnerability of media consumers [23], and in some cases, such as educational purposes, human oversight is necessary to optimize these types of visualizations [24]. The realism perceived by the general public depends on the approach and text prompt used for creating the image to a large extent [25]. When decoding faces, observers may be able to spot fake ones, but they have a harder time discerning real faces from fake faces, and sometimes fake faces seem more real than real faces [26]. In this regard, the question is whether AI-generated images are perceived as more realistic than human-made ones.

Another important and related factor is the credibility of AI-generated content. Research has shown that the context in which information is presented significantly influences how convincing and credible it appears. For instance, studies have found that framing statements within a neuroscience context can affect how scientific reasoning is judged [27]. In educational research, AI research is considered less credible in comparison to neuroscience research or psychology research, and the effect is still evident when individuals have familiarity with the topic [28]. Perceived AI credibility positively affects consumers’ experiences [29]. In democratic societies, the credibility of the news is a highly relevant issue. Several studies have examined how awareness of AI-generated news impacts audiences and readers [30,31]. In any case, deep-learning-based approaches to detect fake images have been developed [32,33,34]. All these approaches place the responsibility for credibility on technological tools. However, it should be desirable for human beings to be capable of determining what is credible and what is not—without relying on yet another AI system to make that judgment for them. For this reason, it is valuable to explore whether individuals attribute more or less credibility to AI-generated images compared to human-made ones, especially when they are unaware of the image’s actual origin.

In this study, we aimed to examine four key aspects related to image interpretation based on the image source (AI-generated versus human-made). First, we investigated whether individuals could accurately distinguish between AI-generated and human-made images. Second, we explored whether the origin of the image influenced the perception of realism. Third, we examined whether credibility was affected by the image’s AI origin. Finally, we assessed whether visual expertise influenced individuals’ performance across these three dimensions: source identification, realism perception, and credibility judgment.

## 2. Materials and Methods

### 2.1. Participants

The final sample was made up of 161 subjects comprising 70 men (43.5%) and 91 women (56.5%). The ages were distributed as follows: 32 (19.8%) from 18 to 24 years, 49 (30.4%) from 25 to 34 years, 30 (18.6%) from 35 to 44 years, 16 (9.9%) from 45 to 54 years, and 34 (21.2%) over 54 years. The subjects were divided into two analysis groups: visual professionals and non-visual professionals. Visual professionals refer to participants with formal education or at least two years of professional experience in fields related to visual media, such as photography, filmmaking, graphic design, visual arts, advertising, or media production. This group was identified based on participants’ self-reported academic background and professional activity in the demographic questionnaire. Non-visual professionals did not meet this criterion. The distribution by groups was 71 (44.1%) in the group of visual professionals and 90 (55.9%) in the non-visual professional group. Participants gave prior informed consent to participate in this study.

### 2.2. Stimuli

**Figure 1:**
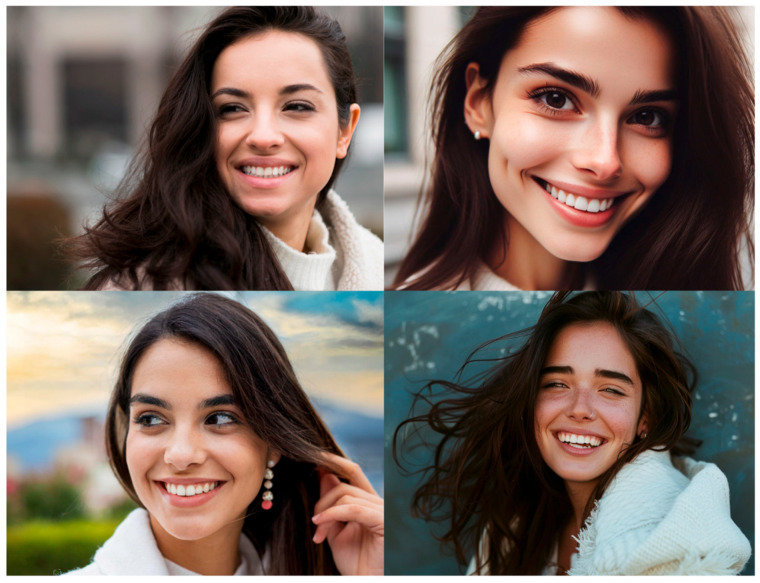
Examples of human portrait stimuli obtained with the same prompt (see Appendix A—Human portrait 1). The top left image was sourced from Freepik (non-AI), the top right was generated using DALL·E 3 (via Bing), the bottom left with Firefly, and the bottom right with Midjourney.

We presented a total of 32 images, divided as follows (see Table 1): human portraits (8), landscapes (8), everyday scenes (8), and detailed objects (8). Each category included 6 AI-generated images and 2 human-made images. In total, participants viewed 24 AI-generated images and 8 human-made images, evenly distributed across the four categories. (see an example in Figure 1). The decision to use a 3:1 ratio of AI-generated (24) to human-made (8) images was intentional and driven by one of our study’s main objectives: to examine participants’ ability to discern AI-generated content across a broader range of examples and styles. This design choice reflects the increasing prevalence and diversity of AI-generated imagery in current visual media, where such content is rapidly becoming more widespread than human-made images. The presentation order of the images was randomized for each participant using the built-in randomization feature of the survey software to control for order effects.

A systematic approach was used for image selection: stimuli of the four above-mentioned categories (human portraits, landscapes, everyday scenes, and detailed objects) were obtained through two distinct strategies. First, for the human-made images, we used a premium account on the Freepik platform (a stock image database), entering a descriptive phrase of our choice into the internal search engine (see Appendix A). Filters were applied to exclude AI-generated content. Note that this search was conducted in March 2024, at a time when AI was not yet deeply integrated into the tool. Once an appropriate image was selected, the second strategy was applied: using the same descriptive phrase, we formulated a detailed prompt to generate comparable images with the three AI tools used in this study—Midjourney, DALL·E 3 (via Bing), and Firefly. To ensure consistency and comparability across conditions, the same prompt was used for all three AI systems within each image category (see Appendix A).

This dual-generation method was selected because it reflects how individuals—whether visually trained or not—typically obtain visual content. When not using AI, a person might either take a photo themselves (with a smartphone or camera) or search for a suitable image using a platform like Freepik. In both cases, the image search is driven by a specific personal or professional need. In this study, the researchers acted as the content-seeking user, establishing the image selection criteria. For the AI-generated images, the same descriptive text used in the stock image database search was applied as a prompt, with the goal of producing a comparable visual output: a single image. The texts used are available in Appendix A (note that Spanish was the language used, but English translations are also reported). All the images were obtained and generated in March 2024.

### 2.3. Variables

#### 2.3.1. Dependent Variables

Three dependent variables were measured in this study: (1) source identification, (2) the perceived realism of each image, and (3) the perceived credibility of each image.

Source identification. This refers to whether the audience perceives the image they are viewing as having been created by AI or by a human. Participants were asked to indicate the origin of the image as “made with AI” or “made without AI”.

Realism. This concept refers to the degree to which the audience perceives the image presented in the study as realistic or resembling reality [35]. Participants were asked to rate the realism of each image using a 5-point Likert scale, ranging from 1 (“not realistic at all”) to 5 (“completely realistic”).

Credibility. This concept refers to the audience’s perception of the reliability and trustworthiness of both the source and the message being conveyed [36]. Participants were asked to rate the credibility of each image using a 5-point Likert scale, ranging from 1 (“not credible at all”) to 5 (“completely credible”).

#### 2.3.2. Independent Variables

The study included two independent variables: (1) the type of image (AI-generated vs. human-made) and (2) participant professionalization (visual professionals vs. non-visual professionals).

Type of image. We used AI-generated images versus human-made images. As explained in the Stimuli section, AI-generated images were created using different tools, all of them in their March 2024 versions. The human-made images were obtained from a repository.

Visual professionalization. As mentioned above (see Section 2.1), visual professionals were defined as individuals who had either formal education or a minimum of two years of work experience in visual fields such as photography, design, advertising, communication, filmmaking, or the arts. Participants who did not meet this criterion were classified as non-visual professionals.

### 2.4. Data Acquisition

A structured survey asking about the dependent variables was distributed online to participants between 16 April and 18 June 2024. The survey presented six sections. In the first one, the consent form was obtained. In the second section, general information such as demography, gender, and visual professional experience was asked about. The third section included the 8 images of the human portraits. The fourth section presented the corresponding images of landscapes. The fifth included the everyday scenes, and the sixth asked about the detailed objects. As explained earlier in Section 2.3.1, each image was accompanied by three questions: one regarding the perceived origin (AI- vs. human-made), one assessing realism (on a 5-point Likert scale), and one evaluating credibility (also on a 5-point Likert scale).

### 2.5. Data Analysis

We performed descriptive and inferential statistics on the data (Supplementary Table S1). First, we analyzed the data altogether to check the dependent variables over the whole sample. Then, we computed segmented analysis divided by the studied topic: human portraits, landscapes, everyday scenes, and detailed objects. We computed linear mixed models (LMMs), taking *p* < 0.005 as statistical significance. We used JASP version 0.19.3 (University of Amsterdam, Amsterdam, The Netherlands, EU) and SigmaPlot 11.0 (Systat Software Inc., San Jose, CA, USA) to run the statistical analysis.

## 3. Results

### 3.1. Source Identification

Overall, we obtained 5152 answers (161 participants × 32 images) distributed as follows: 3864 (75%) images were AI-generated and 1288 (25%) were human-made images. However, 51.24% of images were identified as AI-generated, while 48.76% as human-made. In the case of the AI-generated images, 61.08% were satisfactorily identified as AI-generated and 38.98% were wrongly classified as human-made. In the group of human-made images, 78.03% were correctly identified as human- and 21.74% as AI-generated (see Table 2). In general, these results indicate that AI-generated images are harder to identify than human-made ones.

We then analyzed whether visual professionals performed better than non-professionals at distinguishing between AI-generated and human-made images. For AI-generated images, visual professionals correctly identified them 62.09% of the time, while non-visual professionals did so 60.28% of the time. In the case of human-made images, professionals achieved a correct classification rate of 82.57%, compared to 74.86% for non-professionals. These results indicate a better performance in the visual professionals in both cases.

#### 3.1.1. Human Portraits

We collected a total of 1288 responses related to human portraits (161 participants × 8 images). Of these, 75% (*N* = 966) were AI-generated, while 25% (*N* = 322) were human-made. When participants were asked to identify the source of the portraits, they classified 590 images (45.81%) as AI-generated and 698 (54.19%) as human-made. This indicates that more than half of the images were perceived as human-made, despite only a quarter actually being so.

Segmented analysis (see Table 3) reveals that, among the AI-generated images, only 525 (54.35%) were correctly identified as such, while 441 (45.65%) were misclassified as human-made. In contrast, the majority of human-made images (79.81%) were correctly identified, with only 20.19% mistaken for being AI-generated. These results suggest that, when evaluating human portraits, participants were considerably better at recognizing human-made images than identifying those produced by AI.

#### 3.1.2. Landscapes

We also collected 1288 responses related to landscape images (161 participants × 8 images). As with the previous image types, 75% (*N* = 966) were AI-generated, and 25% (*N* = 322) were created by humans. When asked to identify the source of each image, participants classified 737 (57.22%) as AI-generated and 551 (42.78%) as human-made.

A segmented analysis (see Table 4) reveals that the majority of AI-generated landscapes (65.74%) were correctly identified, although roughly one-third were mistakenly perceived as human-made. Conversely, for human-made landscapes, the pattern was reversed: approximately one-third were incorrectly believed to be AI-generated.

This suggests that, while participants were somewhat more successful at identifying AI-generated landscapes than in other categories, confusion between sources persisted—especially when evaluating human-made content.

#### 3.1.3. Everyday Scenes

Again, we also obtained a total of 1288 answers related to everyday scenes (161 subjects × 8 images). As before, of those, 75% (*N* = 966) corresponded to AI-generated images and 25% (*N* = 322) to human-made images. When asked about the perceived source of the everyday scenes, participants responded that 672 (52.17%) were AI-generated images and 616 (47.83%) were human-made images.

Analyzing the data by the type of source (see Table 5), we find that, in the case of AI-generated images, 37.16% are identified as human-made, while in the human-made images, 19.88% are wrongly attributed to AI. These findings suggest that while participants are relatively effective at recognizing human-made images in the everyday scenes category, they tend to misclassify a significant portion of AI-generated images—indicating a lower accuracy in detecting content produced by artificial intelligence.

#### 3.1.4. Detailed Objects

In the case of detailed object images, we also obtained a total of 1288 answers (161 subjects × 8 images). Of these, 75% (*N* = 966) corresponded to AI-generated images and 25% (*N* = 322) to human-made images. When asked to identify the perceived source of these images, participants classified 641 (49.77%) as AI-generated and 647 (50.23%) as human-made. Segmented analysis (see Table 6) shows that participants correctly identified AI-generated images only in 61.28% of cases. In contrast, they correctly recognized human-made images in 84.47% of cases. These findings indicate that, in the context of detailed object images, participants again demonstrated a greater ability to identify human-made images compared to AI-generated ones.

### 3.2. Realism

**Figure 2:**
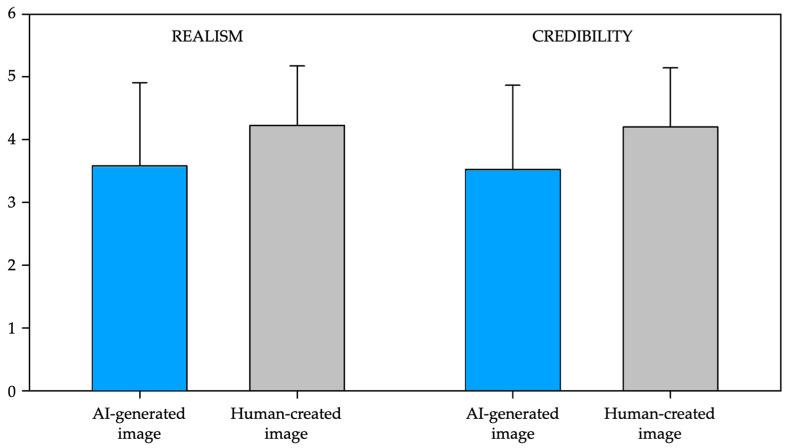
Mean ratings of realism and credibility for the presented images, grouped by image source (AI-generated vs. human-made).

For assessing the realism attributed to the images, first, we determined that the perceived realism was rated (mean ± SD) at a mean value of 3.58 ± 1.326 for AI-generated images and 4.224 ± 0.949 for human-made images (see Figure 2), measured with a 5-point Likert-type scale. This suggested that human-created images were perceived as more realistic than AI images. Visual professionals attributed higher realism to both AI-generated and human-made images compared to non-professionals (Table 7).

Then, we conducted a linear mixed-effects model to examine the effects of professional background (professionals vs. non-professionals) and image type (AI-generated vs. human-made), as fixed-effect variables, on the dependent variable of realism, with ‘subject’ included as a random effect. The analysis revealed a significant main effect of professional background, *F*_(1, 168.62)_ = 8.395, *p* = 0.004, indicating that professionals and non-professionals rated the images differently. There was also a significant main effect of image type, *F*_(1, 4989.00)_ = 537.898, *p* < 0.001, showing a robust difference between AI-generated and human-made images. However, the interaction between professional background and image type was not significant, *F*_(1, 4989.00)_ = 0.524, *p* = 0.469. These findings indicate that while the influence of image source and professional background on realism perception operate independently, both factors significantly shape how realistic participants perceive an image to be—human-made images and evaluations from visual professionals tend to receive higher realism ratings overall.

#### 3.2.1. Human Portraits

We analyzed the effect of the type of image and visual professionalization on realism perception, based on the linear mixed-effect analysis, in each type of content. In the case of human portraits, the LMM revealed that there was not a statistically significant interaction between the source and the visual professionalization of individuals (*F*_(1, 1125)_ = 0.057, *p* = 0.811). Simple main effects analysis showed that the source did have a statistically significant effect on the realism perception of human portraits (*p* < 0.001), and that visual professionalization did not (*p* = 0.094).

#### 3.2.2. Landscapes

In the case of images presenting landscapes, we found that the realism perceived by viewers was not significant in the interaction between the source and the visual professionalization (*F*_(1, 1125)_ = 2.404, *p* = 0.121, LMM), but the main effect of each variable was: visual professionalization (*p* < 0.001) and type of source (*p* < 0.001).

#### 3.2.3. Everyday Scenes

When looking at images related to everyday scenes, viewers did not report significant differences in their perception of realism based on the interaction between the source and the professionalization (*F*_(1, 1125)_ = 0.276, *p* = 0.6, LMM), nor in the isolated effect of visual professionalization (*p* = 0.096). However, the type of source (AI versus non-AI) was relevant (*p* < 0.001).

#### 3.2.4. Detailed Objects

Images presenting detailed objects do not show significant differences in realism perception when looking at the interaction between the source and visual professionalization (*F*_(1, 1125)_ = 0.259, *p* = 0.611, LMM), but the isolated main effects of each studied variable source (*p* = 0.006) and professionalization (*p* < 0.001) do affect the realism perception in a significant way.

### 3.3. Credibility

In terms of credibility, participants rated the AI-generated (mean ± SD) images with a mean value of 3.527 ± 1.338 and the human-created ones with a mean value of 4.199 ± 0.944 (see Figure 2), measured with a 5-point Likert-type scale. These findings indicate that participants attributed greater credibility to human-created images compared to AI-generated ones. Visual professionals rated the credibility of AI-generated images higher than non-professionals, while the opposite happened with human-made images (Table 8).

A linear mixed-effects model was conducted to examine the effects of professional background (professionals vs. non-professionals) and image type (AI-generated vs. human-made), as fixed-effect variables, on the dependent variable of credibility, with ‘subject’ included as a random effect. The results showed a significant main effect of professional background, *F*_(1, 169.08)_ = 5.540, *p* = 0.020, and a significant main effect of image type, *F*_(1, 4989.00)_ = 579.194, *p* < 0.001. Importantly, the interaction between professional background and image type was also significant, *F*_(1, 4989.00)_ = 5.371, *p* = 0.021, suggesting that the difference in credibility evaluations between AI and human-made images varied depending on the participants’ professional background.

#### 3.3.1. Human Portraits

The linear mixed-effect analysis was performed to study the credibility assessed for each type of content. In the case of human portraits, we obtained no significative differences in the interaction effect (*F*_(1, 1125)_ = 0.627, *p* = 0.429), nor in the visual professionalization (*p* = 0.385), but we found that the main effect of source factor was significant (*p* < 0.001).

#### 3.3.2. Landscapes

In the case of landscape images, the credibility ratings showed significant main effects for both factors—image source: AI vs. human (*p* < 0.001) and visual professionalization (*p* = 0.002). However, the interaction effect was not significant (*F*_(1, 1125)_ = 1.887, *p* = 0.170, LMM), indicating that the influence of image source on perceived credibility did not significantly differ between professional and non-professional participants.

#### 3.3.3. Everyday Scenes

For images depicting everyday scenes, the LMM revealed a significant main effect of image source on credibility ratings (*p* < 0.001), but the effect of visual professionalization was not significant (*p* = 0.148). A modest yet statistically significant interaction was found between the two factors (*F*_(1, 1125)_ = 3.887, *p* = 0.049), suggesting that participants’ professional background slightly influenced how the source of the image affected perceived credibility.

#### 3.3.4. Detailed Objects

In the case of detailed object images, both image source (*p* < 0.001) and visual professionalization (*p* < 0.012) had significant main effects on credibility ratings. However, their interaction was not statistically significant (*F*_(1, 1125)_ = 0.373, *p* = 0.541, LMM), indicating that the impact of image source on perceived credibility remained consistent regardless of participants’ professional background.

## 4. Discussion

AI-generated content represents a new paradigm across many creative fields [6]. In some areas—such as advertising or art—being able to tell whether an image was created by a human or by AI may not be particularly relevant. However, in other domains, such as journalism or information sharing, knowing whether an image is real or artificially generated can be crucial. Interestingly, many AIs are currently being developed or trained to detect whether a content was created by another AI or by a human [37]. However, it does not seem like we are training the human eye to make that distinction. Perhaps people with some level of visual experience (as adults) are better able to tell the difference between AI-generated and human-made images—but what about children who are growing up learning to see in an environment filled with unreal images? Will they be able to interpret images based on their source? Recently, during the COVID-19 pandemic, many media outlets used fabricated images of SARS-CoV-2 to illustrate COVID-19-related information, seemingly without concern for the potential impact that unreal visuals could have on the quality and credibility of informative communication [38,39,40].

This study focused on four main aspects of how people interpret images, depending on whether they were created by AI or humans. First, we evaluated participants’ ability to correctly identify the origin of each image. We found that nearly 40% of AI-generated images were misidentified by participants as human-made. This highlights, on one hand, the remarkable progress of AI tools in producing highly realistic visuals—even though the tools used in this study are already a year old and the technology has since advanced further—and, on the other hand, the ease with which AI-generated content can be mistaken for authentic, human-made imagery. Previous research showed that up to half of individuals are unable to distinguish authentic videos from deepfakes [41]. This is particularly concerning in the field of information, as it highlights the increased vulnerability of individuals to misinformation, disinformation, and malinformation [42]. In other, more creative domains, however, it may even seem promising, especially from the perspective of reducing time and investment in image production. Also, it is interesting to note how we perform better at detecting real people versus fake people than in other categories such as landscapes.

Second, we examined how the image source influenced the perception of realism. Overall, human-made images were rated as more realistic than those generated by AI. This indicates that, even when AI-generated images are misattributed to a human source, certain visual cues may still signal to viewers that the image lacks the expected realism of an authentic photograph. Additionally, ratings of human-made images showed lower variability, suggesting a stronger consensus among participants regarding their realism, whereas AI-generated images elicited more diverse judgments. It is important to note that assessing how realistic an image appears to observers is not a new concept introduced by AI. The graphics community has long studied how various visual factors influence the perception of an image as photographic or real, highlighting that a deeper understanding of these factors can significantly enhance the development of image-generation algorithms [43,44]. This is a crucial area for continued research on AI-generated content, as the perception of realism has a clear impact on both brain processing and behavior [45].

Third, we investigated whether the perceived credibility of an image was influenced by its assumed origin. Similarly to the findings on realism, human-made images were generally rated as more credible than AI-generated ones. Moreover, credibility ratings for AI-generated images showed greater variability, suggesting less agreement among participants and a lower overall consensus regarding the trustworthiness of these images. Predicting image credibility is of great interest in many industries whose storytelling relies on visual content [29,34]. In this context, it is important to note that future research could explore the role of specific visual or audiovisual elements in shaping perceived credibility [46].

While our study focused primarily on the roles of realism and credibility in the interpretation of AI-generated versus human-made images, it is important to acknowledge that other attributes—such as artistic quality, creator intent, and emotional impact—also significantly influence how images are perceived and evaluated. Artistic quality can shape esthetic appreciation and affect viewer engagement beyond mere realism [47,48]. Creator intent provides context that may guide interpretation, helping viewers to discern the purpose behind an image and its communicative goals [49]. Emotional impact further modulates perception by eliciting affective responses that can enhance or diminish perceived authenticity and credibility [50,51]. Incorporating these dimensions into future research would provide a broader theoretical foundation and a more nuanced understanding of the complex factors shaping human judgments of AI-generated visual content. Moreover, emotion recognition is a rapidly evolving area of research, with new models and techniques emerging regularly [52,53]. Future studies could build on this progress by incorporating and comparing viewers’ emotional responses to AI-generated versus human-made content, alongside AI-based emotion recognition systems, to explore the potential convergence or divergence between human and machine interpretation.

Lastly, we examined the role of visual expertise in shaping responses across the three dimensions: identifying the image source, assessing realism, and evaluating credibility. Our results showed that visual professionals were more accurate in identifying the origin of images. This aligns with previous studies showing that expertise has an impact on the performance on related tasks [18,19,54,55,56]. Regarding realism, professionals generally attributed higher realism scores to the images—regardless of whether they were AI-generated or human-made—suggesting that non-professionals may have been more cautious or skeptical in their assessments. In terms of credibility, professionals rated AI-generated images as slightly more credible than non-professionals did, while the reverse was true for human-made images. This apparent contradiction warrants further investigation.

## 5. Conclusions

In this study, we aimed to examine how individuals interpret AI-generated versus human-made images. Our findings indicate that people generally struggle to accurately identify the source of an image, highlighting the high level of realism that AI tools are already capable of producing—even in the early stages of this technology. These results suggest that we cannot rely solely on human judgment to discern whether an image is real or AI-generated, raising important concerns for fields where authenticity is critical. A unique contribution of this study is the comparison between visual professionals and non- professionals, revealing notable differences in their ability to assess an image’s source, realism, and credibility—an aspect that adds depth to our understanding of how professional experience shapes image interpretation in the age of generative AI.

## 6. Limitations

An important limitation of this study is the rapid pace at which AI systems are advancing in generating images. The comparisons presented here are based on images created in March 2024, which may already be outdated given the fast development of these technologies. Another notable limitation of this study is the imbalance in the distribution of image types, with 24 AI-generated images compared to only 8 human-made images. This skewed ratio may introduce bias in participants’ perception and classification, potentially affecting the generalizability of the results. While the larger number of AI-generated images reflects the increasing prevalence and variety of such content in today’s media landscape (as we intended to represent), a more balanced dataset would provide stronger and more robust conclusions. Future research should aim to use an equal or more proportionate number of images from each category to further validate these findings and minimize potential biases. Another limitation of this study is its focus on only two dimensions of image interpretation: credibility and realism. While these factors are central to distinguishing AI-generated from human-made images, other important attributes—such as artistic quality, creator intent, and emotional impact—were not considered. These dimensions may also significantly influence how images are perceived and evaluated. Future research should adopt a broader theoretical framework that incorporates these additional factors to provide a more comprehensive understanding of image interpretation in the context of AI-generated content. An additional factor to consider in this study is the lack of control over individual differences that may influence participants’ ability to classify and evaluate images. Factors such as prior exposure to AI-generated content, levels of media literacy, and inherent cognitive biases were not measured or accounted for in our analysis. These variables could significantly affect participants’ perceptions of realism, credibility, and source identification. Future research should incorporate a pre-assessment of media experience and literacy, as well as consider cognitive and perceptual factors, to better isolate the effects of image type and ensure more conclusive results. A further limitation concerns the sample size of 161 participants. Although the total number of answers (5152) may be sufficient to detect medium effects in overall analyses, the sample may be underpowered for more nuanced subgroup comparisons, particularly between visual professionals and non-professionals. Future studies should aim to recruit larger and more balanced samples to ensure adequate power for detecting smaller effect sizes across more complex factorial designs. Also, this study did not include a formal control of image complexity or content beyond thematic alignment, nor did it assess covariates such as AI familiarity or digital literacy, which may have influenced participant performance. Future studies should incorporate such variables to provide more nuanced interpretations of image classification outcomes.

## Figures and Tables

**Table 1:** Distribution of the 32 images used as stimuli.

	AI-Generated Images			Human-Made Images
Topic	Midjourney	Dalle-e 3 (Bing)	Firefly	Freepik
Human portraits	2	2	2	2
Landscapes	2	2	2	2
Everyday scenes	2	2	2	2
Detailed objects	2	2	2	2

**Table 2:** Distribution of the perceived source of the images.

	Original Source	
	AI-Generated Images (*N* = 3864)	Human-Made Images (*N* = 1288)
Classified as AI-generated images	2360 (61.08%)	280 (21.74%)
Classified as human-made images	1504 (38.92%)	1008 (78.26%)

**Table 3:** Distribution of the perceived source of the images of human portraits.

	Original Source	
	AI-Generated Images (*N* = 966)	Human-Made Images (*N* = 322)
Classified as AI-generated images	525 (54.35%)	65 (20.19%)
Classified as human-made images	441 (45.65%)	257 (79.81%)

**Table 4:** Distribution of the perceived source of the images of landscapes.

	Original Source	
	AI-Generated Images (*N* = 966)	Human-Made Images (*N* = 322)
Classified as AI-generated images	635 (65.74%)	102 (31.68%)
Classified as human-made images	331 (34.26%)	220 (68.32%)

**Table 5:** Distribution of the perceived source of the images of everyday scenes.

	Original Source	
	AI-Generated Images (*N* = 966)	Human-Made Images (*N* = 322)
Classified as AI-generated images	608 (62.94%)	64 (19.88%)
Classified as human-made images	359 (37.16%)	257 (79.81%)

**Table 6:** Distribution of the perceived source of the images of detailed objects.

	Original Source	
	AI-Generated Images (*N* = 966)	Human-Made Images (*N* = 322)
Classified as AI-generated images	592 (61.28%)	49 (15.22%)
Classified as human-made images	375 (38.82%)	272 (84.47%)

**Table 7:** Distribution of perceived realism across the images.

Realism (Mean ± SD)			
AI-generated images	3.58 ± 1.326	Visual professionals	3.5 ± 1.37
		Non-visual professionals	3.3 ± 1.39
Human-made images	4.224 ± 0.949	Visual professionals	4.43 ± 0.76
		Non-visual professionals	4.1 ± 1.05

**Table 8:** Distribution of perceived credibility across the images.

Credibility (Mean ± SD)			
AI-generated images	3.527 ± 1.338	Visual professionals	3.47 ± 1.34
		Non-visual professionals	3.29 ± 1.37
Human-made images	4.199 ± 0.944	Visual professionals	3.78 ± 1.25
		Non-visual professionals	4.08 ± 1.03

## Data Availability

The original contributions presented in this study are included in the article/Supplementary Materials. Further inquiries can be directed to the corresponding author.

## Supplementary Materials

The following supporting information can be downloaded at: https://www.mdpi.com/article/10.3390/jimaging11070227/s1, Table S1: Dataset.

## Abbreviations

The following abbreviations are used in this manuscript:

AI	Artificial Intelligence
ANOVA	Analysis of Variance
GAN	Generative Adversarial Network

## Appendix A

The prompts used to obtain the images in the AI tools Midjourney, DALL·E 3, and Firefly, and in the repository of images Freepik.

**Table A1:** Prompts in Spanish and their translation into English.

Topic	Type of Tool	Original Prompt/Text in Spanish	Translation into English
Human portrait 1	Human-made (Freepik)	*Retrato mujer latina sonriente.*	Portrait of a smiling Latina woman.
	AI-generated (Midjourney, DALL·E 3, and Firefly)	*Crea una imagen tipo fotografía realista de una mujer blanca sonriente, una oreja se ve y la otra está cubierta por su cabello oscuro largo, ojos cafés, fotografía en primer plano, exterior, enfoque en ella. Está vestida con un suéter blanco y una chaqueta blanca.*	Create a realistic photo-like image of a smiling white woman, one ear visible and the other covered by her long dark hair and brown eyes, close-up, outdoor photography, focus on her. She is wearing a white sweater and a white jacket.
Human portrait 2	Human-made (Freepik)	*Retrato hombre negro de noche.*	Portrait of a black man at night.
	AI-generated (Midjourney, DALL·E 3, and Firefly)	*Fotografía contrapicada de un hombre negro con camisa negra, pensativo.*	Low angle photograph of a black man wearing a black shirt, thoughtful.
Landscapes 1	Human-made (Freepik)	*Paisaje costero al atardecer.*	Coastal landscape at sunset.
	AI-generated (Midjourney, DALL·E 3, and Firefly)	*Fotografía cálida de una playa tropical, el mar con palmeras de coco en el momento de la salida del sol.*	Warm photograph of a tropical beach, the sea with coconut palm trees at sunrise.
Landscapes 1	Human-made (Freepik)	*Bosque en otoño.*	Forest in autumn.
	AI-generated (Midjourney, DALL·E 3, and Firefly)	*Fotografía de un bosque en otoño, donde hay muchas hojas son naranjas y el sol se ve al fondo.*	Photograph of a forest in autumn, where there are many orange leaves, and the sun can be seen in the background.
Everyday scenes 1	Human-made (Freepik)	*Reunión de trabajo en una sala, una persona está exponiendo.*	Business meeting in a room, one person is speaking.
	AI-generated (Midjourney, DALL·E 3, and Firefly)	*Fotografía de cuatro personas que están en una reunión de trabajo en una sala.*	Photograph of four people in a work meeting in a room.
Everyday scenes 2	Human-made (Freepik)	*Padres e hijos cocinando en casa.*	Parents and children cooking at home.
	AI-generated (Midjourney, DALL·E 3, and Firefly)	*Fotografía de padres e hijos cocinando galletas en casa.*	Photograph of parents and children baking cookies at home.
Detailed objects 1	Human-made (Freepik)	*Bicicleta vintage en un taller de reparación.*	Vintage bicycle in a repair shop.
	AI-generated (Midjourney, DALL·E 3, and Firefly)	*Primer plano detallado de una bicicleta vintage en un taller de reparación.*	Detailed close-up of a vintage bicycle in a repair shop.
Detailed objects 2	Human-made (Freepik)	*Plano detalle de un girasol.*	Close-up of a sunflower.
	AI-generated (Midjourney, DALL·E 3, and Firefly)	*Plano detalle de un girasol.*	Close-up of a sunflower.
